# Nutraceutical Compounds as Sensitizers for Cancer Treatment in Radiation Therapy

**DOI:** 10.3390/ijms20215267

**Published:** 2019-10-23

**Authors:** Marco Calvaruso, Gaia Pucci, Rosa Musso, Valentina Bravatà, Francesco P. Cammarata, Giorgio Russo, Giusi I. Forte, Luigi Minafra

**Affiliations:** Istituto di Bioimmagini e Fisiologia Molecolare-Consiglio Nazionale delle Ricerche (IBFM-CNR), 90015 Cefalù (PA), Italy; marco.calvaruso@ibfm.cnr.it (M.C.); gaia.pucci@ibfm.cnr.it (G.P.); rosa.musso@ibfm.cnr.it (R.M.); francesco.cammarata@ibfm.cnr.it (F.P.C.); giorgio.russo@ibfm.cnr.it (G.R.); giusi.forte@ibfm.cnr.it (G.I.F.); luigi.minafra@ibfm.cnr.it (L.M.)

**Keywords:** nutraceuticals, radiotherapy, cancer

## Abstract

The improvement of diagnostic techniques and the efficacy of new therapies in clinical practice have allowed cancer patients to reach a higher chance to be cured together with a better quality of life. However, tumors still represent the second leading cause of death worldwide. On the contrary, chemotherapy and radiotherapy (RT) still lack treatment plans which take into account the biological features of tumors and depend on this for their response to treatment. Tumor cells’ response to RT is strictly-connected to their radiosensitivity, namely, their ability to resist and to overcome cell damage induced by ionizing radiation (IR). For this reason, radiobiological research is focusing on the ability of chemical compounds to radiosensitize cancer cells so to make them more responsive to IR. In recent years, the interests of researchers have been focused on natural compounds that show antitumoral effects with limited collateral issues. Moreover, nutraceuticals are easy to recover and are thus less expensive. On these bases, several scientific projects have aimed to test also their ability to induce tumor radiosensitization both in vitro and in vivo. The goal of this review is to describe what is known about the role of nutraceuticals in radiotherapy, their use and their potential application.

## 1. Introduction

Despite the relentless development of new and more effective therapeutic strategies in cancer care, improving clinical approaches aimed to personalize anticancer treatments, still represents a primary goal of our time.

The multifaceted nature and variety of tumors, refractoriness to standard chemotherapy, together with their side effects are the first issue of this challenge, nonetheless, the improvement of targeted treatments, which take into account not only of the specific tumor subtype, but also of the genomic features of the patient to be treated.

The so-called tailored therapies include surgery, chemotherapy, immunotherapy and radiotherapy (RT).

While conventional drug treatments often display detrimental effects also on the normal tissue, the principal purpose behind conventional radiation therapy is to deliver a controlled dose of radiation to a defined tumor bulk and to directly hit cancer cells using high- or low-energy photon beams, thus limiting collateral effects to the surrounding irradiated normal tissue area. In contrast to chemotherapy, which has seen a never-ending development of brand new drugs, the concept of RT has remained the same in over a hundred years and advances have mainly affected the technology used in this clinical field [1]. In fact, by the support of imaging techniques coupled with RT, the dose can be delivered more precisely to its target. However, treatment plans always remain the same for each class of tumors not taking into account the molecular profile which characterizes each one of them and that plays instead a pivotal role for the response to RT [2]. Nowadays, several types of neoplasms are treated employing RT and they include breast, ovarian, head and neck, lung, prostate cancers and lymphomas [3].

In recent years, researchers have focused their interest on the mechanisms underlying tumor cell death induced by radiation which is principally caused by the occurrence of genomic damage.

The DNA damage caused by radiation may be direct (through the interaction with the matter in living cells, in particular with the DNA molecules) or indirect (through the production of free radicals generated by water radiolysis). The formation of reactive oxygen species (ROS) as a result of RT is probably the most critical aspect of RT. On one hand, ROS plays a central role in damaging cancer cell DNA leading them to death, on the other hand, it has harmful effects on normal cells. In fact, ROS are normally produced during cell metabolism and a fine-tuned scavenge system is adopted by cells to reduce physiological ROS concentration and their detrimental effects. However, the damage entity of ROS depends on several factors, such as the linear energy transfer (LET) value, the dose and dose rate used during irradiation and on the intrinsic radiosensitivity of the target tissue.

We refer to radiosensitivity as the vulnerability of cells to the detrimental effects of ionizing radiation (IR) and it is commonly accepted that cells with a high proliferate rate (such tumor cells) are more prone to IR damage [1]. Cell radiosensitivity is of paramount importance to achieve a biological effect on the irradiated tumor, and it is different among tissues and can make the difference between a responsive target and an unresponsive target. On this purpose, radiosensitization plays an important role as a neoadjuvant for RT and chemical compounds able to sensitize cancer cells have become a useful tool to improve the efficacy of treatments. Synthetic sensitizers are commonly used as adjuvant in the clinical practice and they can summarily classified in “hypoxic” and “non-hypoxic” sensitizer basing on their ability to restore the physiological levels of intratumoral O_2,_ which levels are notoriously decreased within the tumor bulk. In fact, based on the “oxygen fixation hypothesis”, oxygen can permanently stabilize the radical-induced DNA damage caused by radiation [4]. Thus, the oxygen enhancement effect or ratio (OER), given by the ratio between “radiation dose in hypoxia/radiation dose in air”, describes the IR effects dependent on the presence of oxygen. Nitroimidazoles such as metronidazole, misonidazole, etanidazole and nimorazole are the most common hypoxic sensitizer. In the absence of oxygen, the reduction of the nitro group of nitroimidazoles reacts with DNA radicals caused by IR, stabilizing them as it happens for the “oxygen fixation hypothesis”. These stabilized-adducts lead to DNA strand breaks, thus producing effects on target hypoxic cells [5,6]. However, the neurotoxicity of nitroimidazoles limits their use in clinical practice Also, hyperbaric oxygen is used in association with RT and has shown an enhanced radiosensitization. Halogenated pyrimidines such as bromodeoxyuridine (BrdU) and nucleoside analogs (Bleomycin, doxorubicin, etc.) are instead used as non-hypoxic sensitizers as they interfere with DNA repair thus enhancing DNA breaks caused by radiation therapy [7].

If RT is responsible to have a direct effect on cancer bulk, it elicits, on the other hand, an immunogenic cell death (ICD). ICD is triggered after the release and expression, within the tumor-microenvironment, of tumor-derived bioactive molecules that lead to the activation of resident dendritic cells (DCs) and their further cross-priming of CD8+ T cells which play a pivotal role in tumor eradication [8]. DCs are the most representative group of cells included in the class of antigen-presenting-cells (APCs). APCs are key mediators of tumor surveillance and tumor-killing since they process tumor-associated-antigens (TAAs) and in turn present them to immune effector cells to activate them. For this reason, DCs role can be exploited to increase the efficiency of RT by boosting resident DCs activity with the local administration of immunostimulatory adjuvants [9] or by combining RT with DCs vaccination to improve RT effects [10].

In recent years, the attention of researchers has been focused on the use of natural molecules as a coadjuvant of cancer therapy. Nutraceuticals can easily be recovered and they are less expensive when compared to synthesized drugs. Moreover, their use minimizes all the collateral effects that, together with the side effects of chemotherapy, exacerbate the already poor quality of life of oncological patients. A thorough analysis of nutraceuticals has been performed in regards to their positive effect in association with chemotherapy, however, few data are known about their role as radiosensitizers. For this reason, this review aims to report the literature state of the art of in vitro, preclinical and clinical use of some nutraceuticals in association to RT and to describe how they affect cancer cell sensitivity to IR.

## 2. Curcumin

Diferuloylmethane, better known as curcumin, is the major component of a flowering plant belonging to the ginger family, the Curcuma longa or turmeric. Originally grown in the Asian continent, Curcumin is nowadays used worldwide as a spice to give food a specific flavor and color. Due to its antioxidant and anti-inflammatory properties, it has been used for centuries also as a natural drug by traditional Chinese medicine [11]. Curcumin is a well-tolerated compound, both in vitro and in vivo models used in several scientific studies that report the effects of curcumin on inhibiting cancer cell survival and proliferation. As an example, the MCF-7 breast cancer cell line treated with increasing doses of curcumin [12]. Moreover, curcumin has displayed a general anticancer activity in a wide spectrum of tumors, thus representing a reliable compound not only for treatment in combination with chemotherapy but especially for cancer prevention [13].

Since its capabilities to induce apoptosis and to inhibit cancer cell growth, researchers have sought to determine the ability of Curcumin to radiosensitize target tumor cells. Thus, the literature reports some papers that analyze the effects mediated by Curcumin in several tumor settings.

After exposure to X-ray irradiation (5 Gy), in human immortalized prostate adenocarcinoma cells PC-3) the expression of *TNF-α* which in turn activates *NFκΒ* and *Bcl-2* driven anti-apoptotic signals, is induced. Treatment of PC-3 cells with 2 μM Curcumin before irradiation led to the downregulation of radiation-induced *Bcl-2* expression, cytochrome c release, caspases activation and a block in G2/M cell cycle phase [14]. Thus, Curcumin can radiosensitize prostate cancer cells. Together with prostate cancer, Curcumin mediates the radiosensitization of colorectal cancer cells. Indeed, similarly to PC-3 cells, HCT116 and HT29 human colorectal cancer cell lines treated with Curcumin at a concentration of 25 μM before a single dose of X-ray radiation (10 Gy) showed an enhanced radiosensitivity due to the suppression of both *NFκB* activity and *NFκB*-dependent anti-apoptotic (*IAP2, Bcl-2, Bcl-XL*), inflammatory (*COX-2*), proliferative (*cyclin D1*) and angiogenic (*VEGF*) target genes the expression of *NFκB* target genes [15]. The use of Curcumin has been tested also for glioblastoma multiforme, a highly aggressive malignant glioma for which fractionated RT (60 Gy/30 fractions) is the standard treatment in association with the co-administration of temozolomide. However, the high rate of recurrence is due to radioresistance mechanisms.

In human glioblastoma U87 cell line, the treatment with Curcumin enhanced the effects induced by 3 Gy of X-ray in a dose-dependent manner ranging from 5 to 10 μM, including: reduction of cell viability; arrest of cell cycle in G2/M phase (which is the most sensitive step to radiation); inhibition of two master regulators of tumor progression, the Map Kinases *ERK* and *JNK*, through the activation of *DUSP*-2 which acts as *ERK* and *JNK* phosphatase [16].

An interesting study about the effect of Curcumin as a radiosensitizer was evaluated by our research group in the human non-tumorigenic breast epithelial MCF10A cell line and the human breast adenocarcinoma MCF7 and MDA-MB-231 cell lines. These cells were subjected to combined treatment using 4 doses of X-rays (2, 4, 6 and 9 Gy) and 3 concentrations (2.5, 5 and 10 µM) of free Curcumin (Free-Cur) or Curcumin loaded solid nanoparticles (Cur-SLN). Dose/response curves and dose modifying factor (DMF) values highlighted an increasing radiosensitization effect in a concentration-dependent manner for both the two drugs; MCF7 cells resulted more sensitive to the combined treatment, reaching a DMF value of 1.78 using 10 µM Cur-SLN, while the MDA-MB-231 cells showed to be more sensitive to free-Cur, although a DMF value of 1.38 was obtained with the same concentration of the compound. Trancriptomic and metabolomics approach, with the lowest dose/concentration combination (2 Gy/2.5 µM), revealed a double action of Curcumin, as an anti-oxidant, with a protective role against IR, and as an antitumor compound, given its ability to stimulate autophagy [17].

Encouraging results were also reached for head and neck squamous carcinoma (HNSCC) using both in vitro and in vivo models. In fact, through the regulation of *COX*-2 and *EGFR* crosstalk, Curcumin was able to inhibit *EGFR* phosphorylation and, in turn, to decrease the activation of mitogen-activated protein (*MAP*) kinase, which leads to *COX*-2 expression. This effect was shown in in vitro studies, using the human HNSCC cell line and also in an orthotopic mice model of HNSCC, which were irradiated with a single dose of photon (2–4 Gy) after administration of 15 μM of Curcumin. Moreover, a mouse model of head and neck tumor originated by the injection of HNSCC cells into the middle of the mouse tongue, showed a reduction in both tumor weight and size in tumor-bearing mice treated with the combined regimen of Curcumin and irradiation (respectively 15 μM and 4 Gy) in respect to untreated samples [18].

## 3. Resveratrol

Resveratrol (RV) is a phytoalexin, a natural polyphenol, found in many plants or fruit that are commonly consumed by humans. Its production by plants has the role of protecting them from mechanical injury and the attack of harmful microorganisms such as bacteria and fungi. Beneficial effects deriving from the consumption of RV have been deeply investigated in the last decades after a study conducted in 1992 by Renaud and De Lorgeril also known as the “French Paradox”. The study demonstrated that the moderate consumption of red wine (which is rich in resveratrol) is associated with protective effects against coronary heart disease [19].

Since then, together with cardioprotection, anti-aging effects and cancer prevention of resveratrol have been also described [20,21]. All these effects taken together can appear contradictory, in fact, while cardioprotection or aging are easily reducible to an “anti-oxidant” property, the anticancer one is more reliant on “pro-oxidant” features. This is easily explained with the concept of hormesis, according to which the same compound can have opposite activities that are strongly dependent on the administered dose, mentioning the Swiss physician Paracelsus, “The dose makes the poison” and so is the case of resveratrol which exhibits a dual activity depending on its concentration [22,23].

The anticancer activity of resveratrol has been proven as it negatively regulates many mechanisms including cell growth and cell division (e.g., specifically targeting the *EGF*/*EGF-R* pathway) and mediates cell cycle arrest and apoptosis (through the induction of *CDK*-inhibitors and the regulation of *p53* activation) [24]. In light of its anticancer activity, researchers used resveratrol to test if it plays a role in the radiosensitization of cancer cells as well. Melanoma is the most aggressive type of skin cancer and it is characterized by its high resistance to chemotherapy especially in a metastatic phase. RT has a limited role in the care of melanoma, however, radiation treatment can be used as adjuvant of surgery and chemotherapy to control metastatic spread. In this setting, the combination of 5 Gy γ-irradiation with 50 μM of resveratrol was able to induce, both in murine cell line SW1 and human cell line WM35, a remarkable reduction of the cell survival fraction by clonogenic assays [25]. Resveratrol treatment at 20 μM enhances the effects of IR with doses of γ-rays between 0 and 8 Gy also in non-small cell lung cancer (NSCLC). In contrast to melanoma, radiosensitization may be induced in NSCLC cells through an apoptosis-independent mechanism and it is caused by an increase of ROS generation and DNA double-strand breaks production, which leads to accelerated senescence and cell death [26]. Similarly, to Curcumin, RV has been tested in the SU-2 glioblastoma multiforme cell line treated with X-rays. Interestingly, SU-2 cells pretreated with 75 μM RV and irradiated with doses of X-ray between 0 and 6 Gy) showed a lower proliferation rate compared to cells treated with irradiation alone and a reduction of stemness which is responsible for self-renewal of cancer cells. In addition, an increased expression of LC3-II which plays a pivotal role in autophagy and a reduction of the anti-apoptotic protein Bcl-2 also in a nude mouse model were observed [27]. In another in vitro model of glioblastoma multiforme, such as the U87 MG cell line, the use of RV showed to inhibit the Hypoxia-inducible factor *HIF*-1α. The activation of *HIF*-1α in the tumor context is responsible for reducing both the effect of RT and the uptake of pyrimidine analogs commonly used as chemotherapy agents to kill tumor dividing cells. The combined treatment of U87 cells with 20 μM of RV and 1 μM of iododeoxyuridine (IUdR), and the following irradiation with 2 Gy of γ-rays, induced a decrease in the ability to form cancer cell colonies in vitro and an increase of DNA damage in spheroid cell culture in respect to cells treated with IUdr alone, thanks to the radiosensitizing action of RV [28].

In HNSCC cancers, the over-expression of the signal transducer and activator of transcription 3 (*STAT3*) promoted growth and survival. The phosphorylated form of *STAT3*, moreover, up-regulated the activity of anti-apoptotic proteins, down-regulated the tumor suppressor P53 and gave radioresistance and chemoresistance to the tumor. Surprisingly, in HNSCC FaDu cell line, RV almost abolished the phosphorylation of *STAT3* through the activation of *SOCS*-1 (a negative regulator of *STAT3*), suppressed cell proliferation, induced apoptosis and at concentration of 100 μM it was able to radiosensitize cells irradiated with a 10 Gy dose of photon beam [29].

Despite RT can be considered as a first-line treatment for localized prostate cancer, prostate tumors can become refractory to it. Among the factors responsible for the acquisition of radioresistance, the loss of DOC-12/DAB2 interactive protein (DAB2IP) has been reported. Through the inactivation of the *PI3K-AKT* pathway, *DAB2IP* is involved in the regulation of cell proliferation, survival and apoptosis. To restore radiosensitivity, *LAPC4-KD* and *PC3-KD* radioresistant human prostate cancer cell lines (DAB2IP-deficient) were irradiated with X-rays (2–6 Gy) after treatment with 25 or 500 μg/mL of RV, respectively, and the peanut stem extract (PSE) of Arachis hypogaea, which contains a high amount of RV. The administration of both RV and PSE was able to enhance the effects of IR by inhibiting cell proliferation, by inducing apoptosis through cell cycle arrest and by enhancing and prolonging the kinetics of the IR-induced DNA damage response (DDR), that is lower in DAB2IP-deficient tumors. Similar results were reached in a xenograft mouse model of prostate cancer in which the combination of IR with RV and/or PSE (total dose 12 Gy, 5 or 250 mg/kg respectively) dramatically inhibited tumor growth [30].

Radioresistance is a distinctive feature also of nasopharyngeal carcinomas (NPC). NPCs are more diffused in the Asiatic continent and they are often diagnosed in an advanced and metastatic stage. In order to overcome resistance to IR, RV was used as a pre-treatment (25–150 μM) to X-rays irradiation (0–6 Gy) in the human NPC CNE-1 cell line. Consistently with the data obtained in other cancers, it was found that in NPC-cells RV induced radiosensitization reducing cell viability and colony formation in a dose-dependent manner. In this case, the radiosensitizing activity of RV relied on the inhibition of the phosphorylated form of *AKT* by downregulating *E2F1*. Moreover, the administration of resveratrol in xenograft tumor mice models of NPC, with 50 mg/kg/day and irradiated (4 Gy/day) for consecutive 3 days, significantly reduced tumor volume and weight of mice treated compared to the once treated with IR or RV alone [31].

Recently, the radiosensitive effects of RV have been investigated also in breast cancer. In the MCF-7 human breast cancer cell line, the combinatorial effects of RV, used at the concentration of 0, 10, 30 and 100 μM, together with photon radiation with doses of 1, 2 and 3 Gy, triggered cytotoxic effects, decreased cell proliferation and a cell cycle arrest in the S phase. Surprisingly, such effects were not dependent on the dose of RV used but rather were obtained with the intermediate concentration of 10 μM and 3 Gy irradiation [32].

Summarizing, the administration of chronic and high doses of RV proved to be well tolerated in humans [33], underling its role as a promising adjuvant agent in cancer care.

## 4. Withaferin A

Withaferin A (WA) is a steroidal lactone, a member of a large group of naturally occurring steroids called Withanolides. WA is the first withanolide to be isolated and it was originally obtained in the late fifties as an extract from the leaves of the Indian plant *Withania Somnifera* also known as *Ashwagandha* or Winter Cherry.

Investigations about the antitumoral effects of WA started immediately after its isolation and they proved the growth inhibitory effect of WA on nasopharyngeal carcinoma and osteosarcoma cells [34,35]. Since then, a plethora of studies showed both the in vitro and in vivo benefits of WA as a natural anticancer agent [36].

In 1996, the radiosensitizing effect in vitro of WA in the V79 Chinese hamster lung fibroblast cell line, a cellular model widely used in studies of DNA damage and DNA repair, was reported. In this study, cells were treated with increasing doses of WA (2.1, 5.25 and 10.5 μM) for 1 h and then irradiated with doses from 1 to 8 Gy of γ-rays. WA showed to be well tolerated by cells, the LD_50_ of the drug was 16 μM. At the lowest concentration of 2.1 μM WA did not affect cell viability, however, it was able to mediate a potent cell-killing effect induced by γ-irradiation at a dose of 2 Gy [37]. In the same year, withaferin A was also tested in vivo in the ascitic form of a murine model of mammary carcinoma, Ehrlich ascites carcinoma (EAC). Mice of 6–8 weeks of age were injected intraperitoneally with 10^6^ tumor cells, and 24 h after the injection, they were treated with WA alone or in combination with RT to verify the tumor growth inhibition. To establish the influence of tumor size on the anticancer effects of WA, tumors were allowed to grow up until 10 days before injection with WA. Withaferin A was used at different dose fractions of 5 or 7.5 mg/kg × 8 days after tumor injection, 10 mg/kg × 5 days, 20 or 30 mg/kg × 2 days with or without γ-rays (7.5 Gy). As expected, the administration of WA coupled with a single dose of RT showed tumor growth inhibition and increased the tumor free-survival and the median survival time (MST) of animals. Specifically, mice treated with 5 mg/kg × 8 days produced 40% of tumor-free survival, such percentage increased gradually with the increasing of the dose reaching the 100% of free-tumor survivals and a median survival time of 120 days (a time comparable to 5 years in human) with the maximum dose of 30 mg/kg × 2 days. Similar results were obtained also when WA was administered 5, 7 or 10 days after tumor inoculation, demonstrating that WA can partially overcome the influence of tumor growth. However, at advanced tumor stages, treatment with WA and RT was ineffective. Overall, also in mice, WA was well tolerated and the treatment with RT alone could induce few beneficial results [38]. The in vivo response to γ-irradiation of transplantable mouse fibrosarcoma was investigated. Mice were treated with progressive doses of WA from 10 to 60 mg/kg before a single dose irradiation of 30 Gy and multiple parameters of tumor response were evaluated: The volume doubling time (VDT), defined as the number of days in which the untreated tumor doubles its volume in respect to the treated one; growth delay (GD), the time required by the untreated tumor to reach five times the treated volume; complete remission accounting for total regression and no recidivism within 120 days; partial regression and no response. Fibrosarcomas treated with WA and followed by radiation exhibited a dose-dependent linear increase both in VDT and GD: the increase became significant when the dose was greater than 20 mg/kg, reaching the best efficacy at the concentration of 40 mg/kg. Nevertheless, mice treated with WA in the dose range between 40 and 60 mg/kg showed also a compelling complete remission in 55% and partial remission in 45% of cases [39]. The same experimental conditions (doses from 10 to 60 mg/kg of WA and a single dose of 30 Gy of γ-irradiation) were used to study the effects of WA + RT in fibrosarcoma and to study the effects on melanoma. As expected, the results were roughly identical [40].

In the late years, the attention on the radiosensitizing effects of withaferin A has also been aimed to highlight the pathways which are compromised after WA treatment. Based on the in vitro evidence that WA decreased the viability of the U937 human histiocytic lymphoma cell-line [41], it was investigated if WA could increase the effects of IR in the same cell line model. U937 cells were treated with different concentrations of WA (0, 0.3, 0.5 and 1 μM) in association with increasing doses of X-rays (from 0 to 10 Gy). However, most of the experiments were conducted combining the subtoxic dose of 0.5 μM and 10 Gy of irradiation because, at that condition, WA together with IR can effectively induce almost 40% of cell death and also all the peculiar morphological changes of apoptosis such as cell shrinkage, cytoplasm aggregation and nuclear condensation. Additionally, in respect to the other experimental settings (WA or IR alone), administration of WA followed by IR lead to: Increased levels of ROS production, an increased expression of cleaved *PARP*, down-regulation of *Bcl*-2, the activation of *JNK* and *p38* signaling pathways which are known to be activated by many cellular stresses such as ROS [42]. Almost identical effects observed in the U937 cell line, were obtained by the same group of research in other cell lines, including Caki (renal carcinoma), SK-Hep1 (liver cancer), MDA-MB-231 (breast cancer) and HeLa (cervical cancer) cells exposed to 10 Gy of X-rays after treatment with 4 μM of Withaferin A [43].

## 5. Celastrol

Celastrol or Tripterine, a pentacyclic triterpenoid isolated from the root of the “thunder god vine” (*Tripterygium wilfordii*), is well-known for its anti-inflammatory properties and commonly used in Chinese traditional medicine as a remedy for several pathologies. The discovery of its proteasome inhibitory activity and its antimetastatic capacity elected celastrol as a good candidate for further investigations in the field of cancer biology [44].

The evidence of a chemical compound with related antitumor effects often leads scientists to test it also as a radiosensitizer, that is the case also of Celastrol whose radiosensitizing potential was evaluated both in vitro and in vivo in the PC-3 human prostate cancer cell line. PC-3 cells were treated with two different concentrations of Celastrol (0.2 and 0.4 μM for 1 h before irradiation) and with different doses of X-rays (from 0 to 6 Gy). At the dose of 0.4 μM but not 0.2 it was found that Celastrol significantly enhanced IR-induced cell cytotoxicity and clonogenic cell killing in a dose-dependent manner. Radio-enhancement is often linked to the interference with the radiation-induced DNA damage repair pathways. On these bases, immunofluorescence and western blotting analyses were used to follow the kinetics of appearance and disappearance of one of the markers activated after radiation-induced DNA damage, the phosphorylated form of the histone H2AX (γ*H2AX*). The analysis showed that PC-3 cells, treated with celastrol in combination with IR, were positive for γ*H2AX* for a longer time in respect of cells only irradiated, thus demonstrating that celastrol hampers DNA double-strand break repair. Nevertheless, markers of apoptosis (including cleaved *PARP* and *caspase*-3 activation) were more expressed in cells that underwent combined treatment with celastrol and IR than in cells treated only with X-rays. To test the effects of Celastrol and IR in vivo, a PC-3 xenograft was created in athymic NCr-nu/nu mice. Mice were inoculated with PC-3 cells and when tumors reached 100 mm^3^, they were treated with 1 mg/kg of celastrol (5 days/week for 3 weeks) 1 h before irradiation with a single dose of 2 Gy (5 days/week for 2 weeks). Celastrol showed to be well tolerated and in association with IR, it was able to delay the tumor doubling time compared to IR alone. Moreover, as proved by histological examination, the association of celastrol and radiation therapy significantly improved apoptosis and decreased the formation of new blood vessels (angiogenesis) [45].

Celastrol (0.5 μM) administration was combined with γ-irradiation (range from 1 to 4 Gy) in the NCI-H460 human lung cancer cell line. As expected, cell growth and survival were affected according to the radiation dose delivered, thus the expression of targets involved in radiation sensitivity, such as *EGFR*, *ErbB2*, *Survivin* and *Akt*, were investigated after Celastrol treatment. Except for *Akt*, all the other markers were significantly decreased together with a Celastrol-dependent inhibition of *HSP90* and the consequent destabilization of its client proteins (for example *EGFR*) [46]. Moreover, the radiosensitizing effect of Celastrol on lung cancer cells was proved to be reliant on its quinone methide moiety which enhanced the ROS production after IR [47].

Lastly, the widest analysis to identify potential candidates able to sensitize human lung cancer cell lines, such as A549 and H460, to IR was in silico conducted and through the use of the bioinformatic connectivity map tool, Celastrol was identified as one of the most effective drugs among 30 drugs tested. Based on these preliminary results, the A549 and H460 cell lines were treated with Celastrol 2 μM, 4 h before irradiation with a 6-MV photon beam at different doses (2–10 Gy). Since a clonogenic assay showed that treatment with Celastrol plus IR decreased the survival of both cell lines, the in vivo response to IR after Celastrol administration was evaluated. A preclinical lung tumor model was created injecting the A549 cell line in mice which underwent a combined therapy with Celastrol (2 mg/kg/5 day) and IR (10 Gy) for 12 days. Respectively at days 6 and 12, mice were sacrificed and tumors were analyzed using H&E staining. The efficacy of the treatment was assessed by measuring the mean percentage of the tumor necrotic fraction which correlates with tumor cell death. The assay showed that tumors treated with the combined regimen had larger intratumoral necrotic areas with respect to the ones belonging to groups of mice treated with Celastrol or IR alone [48].

## 6. Ursolic Acid

Ursolic Acid (UA) (also called urson, prunol or malol) belongs, as with the abovementioned Celastrol, to the family of the pentacyclic triterpenoids. It is found in the peel of many fruits such as apples, blueberries and prunes, as well as in many herbs like rosemary and thyme. Despite having been used unconsciously as a beneficial substance for centuries in traditional medicine, the increasing interest in the health effects of natural molecules has recently lead to the description of the pharmacological properties of UA which exerts anticancer, anti-inflammatory and anti-microbial activities [49]. Recently, its radiosensitization activity has also been underlined. In the DU145 human prostate cancer cell line, the treatment with 30 μM of UA for 24 h before γ-irradiation (5 Gy), showed a significant reduction in cell viability respect to untreated cells. The effect was associated with a reduction of cellular volume and condensed or fragmented nuclei, caspase-3 activation, increasing levels of cleaved PARP and DNA fragmentation, typical signatures of apoptosis. The decrease of cell viability, the activation of the apoptotic cascade and an increasing level of ROS generation were shown also in CT26 human colon carcinoma and B16F10 mouse melanoma cells, both treated in the same condition of DU145 cells. Moreover, mice implanted with B16F10 cells and treated with 100 mg/kg and 4 Gy IR for 2 weeks, underwent an inhibition of tumor growth caused by a down-regulation of *Bcl-2* and *Survivin*, proved by western blot analysis of the tumor tissue [50].

Treatment with UA caused a differential effect after exposure of normal or cancer cells to UV. In particular, the human CRL-4000 hTERT-RPE (retinal pigment epithelium used as a control) cell line and the CRL-11147 skin melanoma cells were both treated with 1 μg/mL of UA for at least 8 h before UV irradiation for 5/10 min in order to evaluate the differential UVR-mediated ROS production, cell cycle arrest and cell death. Surprisingly, UA regulated oppositely the UVR-induced oxidative stress in control cells and melanoma cells. In fact, at the same experimental conditions, the DHE assay used to measure the levels of intranuclear superoxide demonstrated an increase in DHE oxidation occurring in cancer cells respect to its oxidized state in control cells, suggesting that UA can act as a photo protector for normal cells and as a photosensitizer for tumors. The cell cycle analysis of irradiated cells showed a cytostatic effect of UA in skin melanoma cells that were enriched in their G1-phase population to the detriment of the S-phase one. Besides, treatment with UA was able to specifically potentiate optical radiation-induced apoptosis and cell death in skin melanoma cells and not in RPE cells, as tested by clonogenic assay and by expression of the apoptotic marker *YO-PRO-1* [51].

Radiosensitizing effects of UA were also highlighted in the BGC-823 human gastric adenocarcinoma cell line. A gastric adenocarcinoma is an aggressive form of cancer for which surgery still represents the best frontline approach, however, often patients are diagnosed in advanced stages and cannot undergo surgery. For those cases with unresectable locally advanced neoplasms, RT is the main alternative to surgery. To determine the radiosensitizing effects of UA, a clonogenic survival assay was performed on the BGC-823 cells treated with UA in the concentration of 0, 6.25 and 10 μg/mL for 24 h and then exposed to increasing doses of 0, 2, 4, 6 and 8 Gy of electron beam radiation. The combination of RT and UA significantly decreased the survival fraction indicating that UA enhanced RT effects in a dose-dependent manner. Compared to cells irradiated or treated with UA only, the combination of 10 μg/mL of UA plus 2 Gy IR showed to induce the arrest of cell cycle in the G1 and G2/M phases and to increase the number of apoptotic cells (positive to *Annexin V* and PI). Moreover, cells treated with UA and RT exhibited higher levels of ROS production (detected by the analysis of DCF-DA mean fluorescent intensity) and a lower percentage of *Ki-67* positive proliferating cells [52].

An interesting study about the effect of UA as a radiosensitizer was carried out on a radioresistant cell line of NSCLC obtained by transfection with a recombinant plasmid expressing a mutant form of *HIF*-1α, the H1299/M-hypoxia-inducible factor-1α. Experiments were performed treating NSCLC cells with 50 or 80 μM/l for 24h before 2 Gy of X-ray irradiation, the cell lines H1229 and H1229 transfected with an empty plasmid were used as a comparison. Intriguingly, results showed that when irradiated after pretreatment with UA, NSCLC cells and especially *HIF-1*α-expressing cells were more sensitive to irradiation respect to the other cell lines. Such sensitization was correlated also with increasing levels of DNA damage assessed by the analysis of the formation of micronuclei, remarkable diminished levels of endogenous glutathione (considered as one of the most important scavengers of ROS), increasing ROS production and a marked inhibition of HIF-1α protein levels [53].

## 7. Zerumbone

Zerumbone (2,6,10-Cy-cloundecatrien-1-one, 2,6,9,9-tetramethyl-,[E,E,E]-) (ZER), a monocyclic sesquiterpene compound, is a cytotoxic component isolated from rhizomes of *Zingiber zerumbet Smith* [54,55]. According to its phytomedical properties, it has been used since ancient times as a condiment in food and herbal medicine in eastern countries [56]. It has been also shown to have anti-inflammatory, anti-proliferative and antitumor properties in several tumor types, such as breast, pancreas, colon, lung and skin [57,58,59]. Moreover, in the last years, some studies revealed that Zerumbone plays a sensitizing effect on tumors after treatment with IR [60], being involved in the regulation of DNA DSBs’ repair induced by IR and in the regulation of cell cycle and apoptotic pathway [61].

As regards radiosensitizing effects of Zerumbone, some researchers described that the combined treatment with ZER (10 µg/mL) and γ-rays irradiation (range of 5–10 Gy) increased the radiation-induced and the heat shock protein (HSP)-mediated cell death in the NCI-H1299 lung adenocarcinoma cell line 48–72 h after irradiation. In addition, ZER enhanced the cleavage of *Caspase* 3 and *PARP*. Furthermore, the same combined treatment, used in in vivo nude mice models after grafting of NCI-H460 and NCI-H1299 non-small cell lung cancer (NSCLC) cells, inhibited the binding of HSP27 to apoptotic molecules such as *Cytochrome* c or *PKCδ* [60].

In the U87 MG and U373 MG human glioblastoma cell lines, pretreatment with ZER followed by progressive doses (range of 0–4 Gy) of X-rays induced inhibition of Gli-1 expression, that usually correlates with metastasis and tumor relapse. In particular, it has been seen that D0 (radiation dose with 37% survival) values, for both cell lines, were lower when treated with zerumbone compared to the control (U87 MG: 3.4 Gy vs. 4.3 Gy; U373 MG 2.6 Gy vs. 4.1 Gy) [62].

In addition, pre-treatment of PC3 and DU145 human prostate cancer cells with ZER (10 µM) before administration of different doses of IR (0–6 Gy) decreased cell survival, abrogated the expression of γ-*H2AX* and reduced the expression of phosphorylated *ATM*, *JAK2* and *STAT3* proteins, all of them involved in the DNA damage repair pathway [63].

In the HCT116 and HT29 colon-rectal cancer cell-lines, different concentrations of ZER (5, 10, 25 µmol/L) were added 4 h before and 3 h after γ-rays irradiation (2, 4, 6 Gy). In particular, treatment with 10 and 25 µmol/L, radiosensitized both cell lines inducing apoptosis and enhancing the radiation-induced G2/M arrest at 2 and 4 Gy. The evaluation of γ-*H2AX* foci showed that their number remained higher until 24 h post IR treatment. Furthermore, in both cell lines, pre-treatment with ZER decreased the radiation-induced expression of two proteins involved in DSBs repair, p*ATM*^Ser1981^ and DNA-*PKCs*, and it depleted the levels of intracellular Glutathione (*GSH*) [64].

## 8. Caffeic Acid Phenethyl Ester

Caffeic acid phenethyl ester (CAPE) is an active component of honeybee propolis, a phenolic compound and a structural derivative of flavonoids [65]. It has antiviral, bactericidal, anti-inflammatory and antioxidant properties, and it has been proved to be more toxic for cancer cells than normal ones [66]. In particular, CAPE can change the redox state by perturbing the activation of *GSH* and inducing apoptosis in transformed cells. It has been also reported that CAPE could potentiate the effect of RT in several types of cancer [67].

It was shown that CAPE might enhance radiation-induced cell cycle arrest or apoptosis in medulloblastoma cells. In the human medulloblastoma Daoy cell line, the combined treatment with CAPE (3, 10, 30 µM) and IR (2 Gy), showed an enhancement in ROS production and significant inhibition of *NF-kB* activity. Also, levels of apoptosis and DNA fragmentation increased, with a parallel down-regulation of Cyclin B1 protein expression [68].

In the same cell line, other researchers described that pretreatment with CAPE (0.1–10 µM) for 24 h before exposition to γ-rays irradiation at various doses (0, 2, 4, 6, 8 Gy), induced a reduction of the cell survival fraction in a concentration-dependent manner, with SF values of 100, 88.5, 56.1, 24.7 and 0%, respectively. Moreover, these data showed that CAPE inhibited cell-cycle progression by arresting cells in the S-phase [69].

The radiosensitizing effect of CAPE was also shown in mouse CT26 adenocarcinoma cells, both in vitro and in vivo. In CT26 cells, pretreatment with CAPE (2 µg/mL) before X-rays irradiation (2, 4, 6 and 8 Gy) decreased the cell survival rate and reduced the *NF-kB* activation. Also, in mice bearing CT26 tumor cells, pretreatment with 10 mg/kg CAPE followed by IR (10 Gy), induced a marked inhibition of tumor growth and volume [70].

X-rays irradiation with doses of 2, 4, 6, 8 Gy) on the MDA-MB-231 and T47D breast cancer cell lines, after treatment with CAPE (1µM) for 72 h, decreased both survival rate of MDA-MB-231 (at 6 and 8 Gy) and T47D (at 2 and 4 Gy) cells. In particular, this combined treatment delayed the DNA repair process for up to 60 min after exposure. In addition, by the comet assay, it was shown that the % DNA in tail, directly proportional to DNA damage, reverted almost to the control value (8.9) 120 min after exposure on the MDA-MB-231 cells, but remained higher (14.9) for up to 120 min on the T47D cells [71].

Finally, the use of CAPE was tested also in the human A549 lung cancer cell line. Treatment with CAPE at various concentrations (0, 2, 4 and 6 µg/mL) for 1 h, combined with different doses (0, 2, 4, 6 and 8 Gy) of X-rays irradiation, were associated with a reduction in the cell survival rate, mostly observed at higher doses of CAPE and IR [72].

## 9. Emodin

Emodin (6-methyl-1,3,8-trihydroxyanthraquinone) is a natural phenolic compound extracted from the roots and rhizome of several plants, such as the traditional Chinese herbs *Rheum palmatum*, *Polygonum cuspidatum* and *Cascara buckthorn* [73,74]. Emodin shares a similar molecular structure with DMNQ (2,3-dimethoxy-1,4-naphthoquinone) and mitochondrial ubiquinone, and it could be qualified as endogenous ROS generators because of its property of transferring electrons [75]. It has antibacterial, antiviral, anti-inflammatory and anticancer effects [76,77]. Emodin’s mechanism of action in inhibiting the development of cancer remains barely elucidated, but its antitumor action has been observed in leukemia, breast cancer, colon cancer, lung cancer, etc. [78], also in association with RT.

It has been proved that exposure of CNE-1 NPC human nasopharyngeal carcinoma cell line to Emodin under hypoxic conditions enhanced their radiosensitivity. In particular, treatment with 3.9 and 7.8 µg/mL of emodin, 24 h before irradiation with 2 Gy IR, caused an increase in the apoptosis ratio (%), with a value of 25–21, and the arrest of the cell cycle in G2/M phase. Furthermore, under hypoxic conditions, this combined treatment leads to an increase in the relative content of ROS (161–149%) and to a decreased expression of *HIF-1α* mRNA and protein. Furthermore, in vivo experiments on CNE-1 xenograft model showed that combined treatment with Emodin and 2 Gy IR caused a tumor growth delay of 6.90 and 9.15 days, for low (4 mg/kg) and high dose of Emodin (12 mg/kg), respectively [79].

Interesting results in other cancer cell lines were also found. In HeLa cervical cancer cell line, treatment with different concentrations of Aloe Emodin (AE) (0, 50, 100 and 200 µM) before the exposition to different doses of X-rays irradiation (0, 2, 4, 6, 8 and 10 Gy), induced an alteration of some radiobiological parameters of the dose survival curve. In particular, there was a decrease in the mean lethal dose (D0), in the quasi-threshold dose (Dq), in the extrapolation number (N) and the daily fraction dose of 2 Gy in clinical practice (SF2), and an increase in the sensitizing enhancement ratio SER(D0) and SERDq in a concentration-dependent manner. The analysis of cell cycle distribution and apoptosis, after treatment with 50 µM AE and 4 Gy IR, showed an increase in the number of cells in the G2/M phase and a sub-G1 peak at 24, 48 and 72 h. In addition, this combined treatment increased the expression of *cyclin B,* γ-*H2AX* and *ALP* activity [80].

The combined treatment with γ-rays (10 Gy) and AE (10 µM) induced a significant decrease of growth and viability on the human HepG2 hepatocellular carcinoma cell line, also under hypoxic conditions. This treatment induced a greater increase of both G2/M and apoptotic population, tested by an increase of expression in cleaved *PARP*-1 levels, a decrease in the expression of *HIF*-1α and its target genes, such as *JMJD1A* and *JMJD2B*, involved in hypoxia-induced radioresistance [81].

The same experimental approach was carried out using the FSa p53 mutant (Mut) murine sarcoma cell line. Exposure to 50 µM AE before irradiation with 2 Gy X-rays increased the nuclear Survivin levels and in the decrease of the nuclear transport protein *CRM*-1, which is involved in the export of Survivin from the nucleus to the cytoplasm [82].

In the CN1-E nasopharyngeal carcinoma cell line, pretreatment with 10 mg/L of GXHSWAQ-1 (a synthetic compound created on the basis of the chemical structure of Emodin) for 24 hrs before X-rays irradiation (2 Gy), caused a damage in the integrity of mitochondrial structure, with swelling and/or matrix compartments vacuole formation and a collapse in the transmembrane potential. A proteomic analysis showed that, in the radiosensitized group, *Rac1* and *CDC42* protein expression, whose decrease correlates with a high invasive potential of cancer cells, were higher, while *CDH1* protein expression, usually known as a potent suppressor of radiosensitization, was significantly lower [83].

## 10. Flavopiridol

Flavopiridol is a synthetic flavone derived from *Dysoxylum binectariferum*, a plant used in Indian medicine [84]. This molecule inhibits the Cdks activities arresting the cell cycle (G1/S or G2/M phases) [85]. The cell cycle arrest was observed in many histotypes of cancer [86,87]. This effect on the cell cycle was analyzed and confirmed in several experimental tumor models, such as chronic lymphocytic leukemia, squamous cancer and breast cancer cells. Moreover, it plays an anti-proliferative effect through the transcriptional suppression activity of genes involved in the proliferation pathways [88]. In several models in vitro and in vivo, it was demonstrated that Flavopiridol stimulates apoptosis [87], inhibits angiogenesis [89] and increases the chemotherapeutic effects [90].

A study in vitro and in vivo evaluated radiosensitivity effects of docetaxel and Flavopiridol following the radiation exposure on the lung carcinoma. The H460 human lung carcinoma cell line was treated with this sequence: docetaxel (10 µM), γ-irradiation (0–5 Gy) and Flavopiridol (120 µM), while H460 cells were inoculated in nude mice which were treated with docetaxel (2.5 mg/kg), γ-irradiation (2 Gy) and Flavopiridol (1.25 mg/kg). Researchers performed different experiments by testing different treatment combinations and have observed after treatment an increase of radiation effects in vivo and in vitro with an arrest of the cell cycle (G1 and G2/M) [91].

In the esophageal squamous carcinoma, the treatment of three cell lines, such as TE8, TE9 and KE4, with or without Flavopiridol (0.05 nmol/L) before X-ray irradiation (2–10 Gy) induced a decrease of the levels of cyclin D1, Rb in all cell lines and of Bcl-2 protein in the KE4 cells. The experimental data demonstrated the enhanced radiosensitivity of cell lines and suggested that the use of Flavopiridol at a low dose could represent an efficacy therapeutic approach against the esophageal squamous carcinoma [92].

Besides, the Eca109 esophageal squamous cancer cell line was treated with gradient concentrations of flavopiridol for 48 h (0–517.5 nM) and X-ray irradiation (0, 2, 4, 6 and 8 Gy). The results highlighted a decrease of the Cyclin D1 expression level, enhancing the percentage of cells in the G2/M phase. Moreover, *Caspase*-3 and *Bax* proteins increased in Eca109 cells, while *Bcl*-2 expression decreased. Therefore, the Eca109 cells, treated with Flavopiridol and radiation, showed a more radiosensitivity [93].

The radiosensitizing effect of Flavopiridol was demonstrated in the SEG-1 esophageal adenocarcinoma cells. The in vitro results obtained highlighted that cells treated with Flavopiridol (300 nM) for 24 hrs before or 7 h after γ-radiation (2–6 Gy) showed greater radiosensitivity respect to the control [94]. This mechanism is multiple and is characterized by the inhibition of some CDKs, the redistribution of the cell cycle with an accumulation of SEG-1 cells in G1 and G2 phases and induction to apoptosis. Moreover, in the xenograft mouse model, a single dose of Flavopiridol (15 mg/kg) in combination with γ-radiation (15 Gy) was sufficient to determine an enhance of the SEG1 tumor tissue response to irradiation. The results showed that the Flavopiridol administration before (4 h) irradiation is more effective than after (7 h) [94].

A research group analyzed in glioma and cervical tumor cells the potential involvement of *p53* and *Bcl-2* in radio-sensitivity mechanisms, following treatment with Flavopiridol [95]. These proteins are known to be involved in cell cycle regulation, DNA repair and apoptosis. Previous works showed that the difference in the expression levels of *p53* and *Bcl*-2 proteins can lead to radioresistance phenomena in several cancers [96,97]. A172 glioma and HeLa cervical tumor cells were stably transfected with plasmids containing mutated forms of *p53* (A172/mp53) and *Bcl*-2 (HeLa/bcl-2) genes, respectively. Cell lines were treated with Flavopiridol (0.0125 μM for A172 and 0.1 μM for HeLa) for 24 hrs after irradiation (2–8 Gy) and cell viability and potential DNA damage were evaluated. The experimental data showed that cells that contained mutated *p53* or overexpressed *Bcl*-2 were more radioresistant than wild-type, confirming a potential key role of these proteins in the mechanisms of radioresistance. Furthermore, the treatment with Flavopiridol increased the cytotoxic effects of radiations in the transfected cells compared to the untreated ones. It has been hypothesized the presence of a common pathway or targetable molecule would be valuable in order to determine the radiosensitizing effect of Flavopiridol mediated by *p53* and *Bcl-2*. This molecular target could be the *RAD51* protein, involved in the regulation of the repair processes following DNA damage due to radiation exposure [98,99]. This protein interacts with p53 and is inhibited from the high expression levels of *Bcl*-2 [100]. Probably, the Flavopiridol action occurs interfering with the molecular interaction between *RAD51*, *p53* and *Bcl*-2 and thus in the repair mechanisms. However, this hypothesis needs further clarification. It has been suggested that the use of Flavopiridol as a radiosensitizer molecule could be useful for the treatment of tumors with an altered status of *p53* and *Bcl*-2 [95].

The radiosensitizing effects of Flavopiridol were evaluated in vivo by inoculation of GL261 glioma cells in murine models. The tumor control/cure dose of radiation assay (TCD50) was performed to measure the dose of γ-radiation (65 Gy) required to treat 50% of local tumors. The combined treatment of γ-radiation (5 Gy), fractionated for 10 days with the administration of Flavopiridol (5 mg/kg) in the murine models showed a decrease in cell growth and a greater sensitivity to radiation. Moreover, it has been hypothesized that the anti-angiogenic effect exerted by the Flavopiridol inhibits the *HIF*-1 pathway and thus the *VEGF* factor [101] that is closely associated with the radioresistance mechanisms [102].

The OCA-I ovarian carcinoma cells treated with Flavopiridol (300 nM) for 24 h and γ- radiations (1–6 Gy) exhibited an increase of the radiosensitivity compared to control. The results showed an expression decrease of *Ku70* and *Ku86* proteins involved in the repair mechanisms [103] a redistribution of the cell cycle with a greater accumulation of these cells in phases of cell cycle more radiosensitive, as G1 and G2 [104,105] and an inhibition of cyclin/cdk complexes through the attenuation of the phosphorylated form of *Rb*, blocking cell cycle [106]. Moreover, the Flavopiridol could repress gene transcription process through the inhibition of *Cdk*-9 that is involved in DNA repair with *Cyclin T* [107] and *K* [108] and regulates RNA polymerase II activity through the formation of a cyclin-complex [107]. These results strengthen the use of Flavopiridol as a radio-sensitizing molecule [109].

The cytotoxic effects of Flavopiridol were evaluated on the HeLa human uterine cervix cancer cell line exposed to irradiation with X-rays (0, 2, 5 and 10 Gy,) and treated with Flavopiridol (IC50 = 80 nM). In particular, it has been observed that a considerable reduction of the cell survival fraction occurred by treating the cells for 24 h after irradiation, while no significant results were observed in cells treated before irradiation or simultaneously [110].

The treatment of the DU145 and PC3 human prostate cancer cell lines with Flavopiridol (60–90 nM) at three different times (1, 6 and 24 h) after X-ray irradiation (2 Gy) showed in both cell lines a higher number of *γH2AX* foci in cells treated for 24 h with Flavopiridol and radiation, compared to cells treated with irradiation alone. The increased expression of *γH2AX* could be an index of inhibition of the mechanisms of DNA repair Flavopiridol-mediated [111].

A study performed on the zebrafish model evaluated the Flavopiridol radiosensitizing effect, also verifying if it occurred through the *Cyclin D1* inhibition, as previously demonstrated in ovarian cancer cells [109]. The results above confirmed the radiosensitizing role of this molecule. Indeed, the embryos treated with Flavopiridol (500 nM) before irradiation with γ-rays (10–40 Gy) displayed reduced viability compared to the control and exhibited a morphological alteration of the dorsal tail, due to exposure to radiation. This change appears to be dose-dependent. Moreover, the micro-injection in the embryos of the antisense hydroxylprolyl-phosphono peptide nucleic acid oligomers (0.5 pmol), which down-regulated *cyclin D1*, and irradiation determined not only a reduction in viability but also the manifestation of the same phenotypic characteristics observed in the embryos flavopiridol-treated. These data are proof of the *cyclin D1* involvement in the radiosensitizing mechanism flavopiridol-mediated [112].

## 11. Berberine

Berberine, an alkaloid component extracted from several medicinal herbs, including Huang Lian, is characterized by low toxicity. Therefore, it is widely used as a drug for gastrointestinal discomfort in China and has been tested in clinical trials for type 2 diabetes mellitus [113,114] and on hypercholesterolemia [115]. Research has demonstrated that berberine has an antitumor activity for a wide variety of cancer cells [116,117,118,119,120,121,122]. This effect often occurs through inhibition of the cell cycle progression and the promotion of apoptosis. A study showed that Berberine possesses a radiosensitizing feature shown in lung cancer cells [123].

Liu Z. et al. have previously reported that Berberine may inhibit cancer cell proliferation by inducing DNA double-strand breaks in osteosarcoma cells [122]. In a subsequent study, the researchers have demonstrated that Berberine at low concentrations can significantly radiosensitize the esophageal cancer cells (ESCC). The cancer cell lines (KYSE30, KYSE450, KYSE410, EC109 and TE-1) treated with Berberine (15 µM) for 24 h and X-ray irradiation (2–6 Gy) exhibited a major sensibility to the radiation exposure. The experimental data showed that this effect is mediated from the downregulation of *RAD51* involved in the repair of DSBs. The overexpression of *RAD51*, found in human ESCC tissues, suggested the potential use of this protein as a biomarker associated with the radiation response. Moreover, at radiosensitizing concentrations Berberine not determined effects or downregulation of *RAD51* in non-malignant cells. Therefore, it is supposed that the radiosensitizing effect of Berberine may be specific to the ESCC cells [124].

Another study evaluated the radiosensitivity effects of berberine on the hypoxic ECSS cell lines (ECA109 and TE13). The ECSS cells in hypoxic conditions were treated with a low concentration of Berberine (5 μM and 15 μM) for 24 h and irradiated with X-rays (1–9 Gy). The hypoxic cells were more radioresistant respect to the growth of the cells in normal conditions, but the treatment with Berberine sensitizes these cells to the radiation exposure. Therefore, the experimental data showed the decrease of the survival fraction of hypoxic cells following the berberine treatment and the inhibition of *HIF*-1α, associated with the radio-resistance phenomenon [125] and *VEGF*, a protective factor of the endothelial cells from radiation damage [126]. The in vitro results were confirmed through the inoculation of ECA109 in the nude mice, treated with Berberine (5 mg/kg) for two days before the X-ray radiation (8 Gy). The data in vitro and in vivo highlighted that berberine increased the radiosensitivity of ESCC cells and xenografts, and this effect was associated with the inhibition of *HIF*-1α and *VEGF* expression [127]. The same experimental design in vitro and in vivo was applied on the prostate cancer cells -LNCaP, DU-145- and the nasopharyngeal carcinoma (NPC) cell lines CNE-1, CNE-2. The same results were observed confirming the importance of the *HIF*-1α and *VEGF* expression in the mechanism of radiosensitivity Berberine-mediated in prostate cancer [128] and NPC [129].

Another human prostate cancer cell line (PC-3) was treated with berberine (30 μM) for 24 h and exposed to γ-irradiation (4–6 Gy) showed a high apoptotic index. It was supposed that this molecule induced the apoptosis mechanism through the pathway generation of the ROS in prostate cancer. Moreover, the study highlighted that the combined action of Berberine and radiation on the prostate cancer cells determined the deregulated expression of several molecules, involved in the apoptotic process, cell cycle and radio -sensitizing/-resistant mechanisms, such as *Bcl-2* [130], *NF-Κb* [131], *p53* [132], *p38* and *JNK* [133]. Therefore, the researchers suggested the use of Berberine as a radiosensitizing molecule to enhance the effect of RT [134]. Moreover, it was observed that the CNE-2 cells (NPC), treated with variable doses of Berberine (25–100 µmol/L) for 24 h and γ-irradiation (4–8 Gy) were characterized by a decrease of the mRNA and by a down-regulation of the protein expression of *Sp1*, a transcription factor associated to the tumoral migration [135,136,137] and correlated to the tumoral invasion and radioresistance in NPC patients [138]. This molecular process involved, also, the selective inhibitor of *Sp1* (Mithramycin A), which is enhanced in the CNE-2 cells, treated with Berberine and irradiation. These experimental results demonstrated the involvement of *Sp1* and Mithramycin A in the radioresistant pathway in NPC and highlight the potential role as therapeutic targets [139].

Finally, the radiosensitizing action of Berberine was evaluated in breast cancer models. The breast cancer cells MCF-7 and MDA-MB468 were treated with berberine (15 μM) and exposed to variable doses of X-rays (1–4 Gy). The experimental results showed that the treatment with Berberine determined the cell cycle arrest, the inhibition of the activation of the repair mechanisms of X-ray-induced DSBs, through the evaluation of *γH2AX* foci, and the downregulation of *RAD51* [140].

## 12. Genistein

Genistein, a soy isoflavone, inhibits cell proliferation and thus enhances apoptosis by inhibiting the activity of tyrosine protein kinases and DNA topoisomerase II. Moreover, these molecules promote the DNA repair mechanism, performing an anti-angiogenic and antitumor effect [141,142] Several studies have demonstrated that Genistein inhibits in vitro the growth of several cancer cells, including lymphoma, melanoma, neuroblastoma, breast and prostate cancer cells [143]. Experimental results showed that the combined treatment with Genistein (40 μmol/L) and γ-irradiations (4 Gy) inhibited significantly the cell growth of the cervical cancer cells (Hela) and increased the radiosensitivity through a down-regulation of Survivin, which inhibited caspase 9 and blocked the apoptotic pathway [144]. This inhibitor was absent in normal differentiated tissues, while it was highly expressed in malignant tumors [145]. Clinically, high levels of Survivin have been associated with a reduction in survival and an increase in relapse and resistance to therapy [146,147]. Therefore, it was suggested the use of Genistein to reduce the IR therapeutic dose and the possible adverse reactions correlated to RT [148]. Based on previous studies showing the ability of Genistein to inhibit the growth of cervical tumor cells in vitro [149], Yashar et al. [150] evaluated the possible role of this compound as a radiosensitizer in other epithelial cervical cancer cell lines (CaSki and ME180) with two different spectra of HPV infection. Both cell lines were treated with Genistein (1–80 µM) for 48 h before photons radiation (200, 500 or 800 cGy). At 40 µM, less than 5% of ME180 cells survived all the radiation doses, while an increase in radiosensitization in CaSki cells was seen only at 500 and 800 cGy with all Genistein doses (in a dose-dependent manner). Additional studies showed as Genistein acts as a radiosensitizer blocking cell cycle in G2/M, specifically in ME180 cells, and moreover, in both cell lines, leading to a dose-dependent induction of Cytochrome c by a reduction of *Mcl-1* and total *AKT*, suggesting an involvement in the apoptotic pathway [150]. Encouraging results in cervical tumor cells were also obtained by other researchers. Combined treatment of different doses of Genistein (0–200 µM) and γ-irradiation (10 Gy) in CaSki and human normal keratinocyte HaCaT cells, showed that the pretreatment with Genistein rendered CaSki cells hypersensitive to the death effect of IR, revealing an induction of apoptotic bodies. In particular, it was observed the inhibition of cell proliferation with an accumulation of cells blocked in the G2/M transition, the increase in the expression levels of *p53*, *p21* and *Cdc2*-tyr-15-p and the decrease of the *cyclin B* levels. Apoptosis induction was shown to be associated with *cytochrome c* release, cleavage of *caspase-3* and *-8*, inhibition of the anti-apoptotic *Bcl-2* expression and enhancement of pro-apoptotic *Bax* expression, upregulation of intracellular ROS and downregulation of *COX-2* expression and *PGE2* production [151].

A study investigated the radiosensitizing effect of Genistein on breast cancer cells with different estrogen receptor (ER) status. Human breast cancer cell lines MCF-7 and MDA-MB-231 were treated with Genistein (5–20 µM) and irradiated with X-rays (4 Gy). The experimental data showed the increase of DNA damages, the arrest of the cell cycle at the G2/M phase, through up-regulation of phosphorylation of *ATM*, Chk2, *Cdc25c* and *Cdc2*, and the enhancement of radiosensitivity through a mitochondria-mediated apoptosis pathway [152].

The radiosensitizing effect of Genistein was tested on the non-small cell lung cancer (NSCLC) cells (A549) and the normal lung fibroblast cells (MRC-5). Cells were treated with Genistein (10 µM) for 48 h and then irradiated with X-rays (4 Gy). The experimental results showed that in A549 cell line the Genistein enhanced the cellular damages from oxidative stress, increasing ROS and decreasing *GSH*, an antioxidant factor [153]. It was observed that Genestein influenced the DNA methylation status [154,155]: in particular, researchers showed that this treatment involved inhibition of methylation in the promoter region of *Keap1*, causing an increase in the transcription/translation levels of this gene. This molecular modification led to the inhibition of *Nft2*, an antioxidant factor [156], and the deregulation of the oxidative system [157]. The result of this process was an increase in the apoptotic levels and a more radiosensitivity of the A549 cells. Furthermore, it is interesting that this mechanism involving the *Keap1*/*Nft2* pathway was opposite in the normal lung fibroblast MRC-5 cells. Therefore, it has been observed that these normal cells treated with Genistein were characterized by an activation of *Nft2*, which determined a greater synthesis of antioxidant enzymes (such as *GSH*) and a decrease of the apoptotic rate and the radiosensitivity. These results showed not only the radiosensitizing effect of Genistein, but also the specificity of this effect on the A549 cells compared to MC5 cells [158]. In another study, the NSCLC cells (A549, Calu-1, H1975 and H460) were treated with 30–60 µM of Genistein for 24 h and exposed to IR (4 Gy). This combined Genistein–IR treatment led to a decrease of the cytoplasmic levels of *Bcl-x*, a known anti-apoptotic factor associated with the radioresistance of lung cancer patients, as demonstrated in some works [159]. Furthermore, *Bcl-x* was also involved in the regulation of autophagy through the molecular interaction with the Beclin-1 protein [160,161]. Summarizing, the authors suggest that the Genistein has a potential radiosensitizing effect in the NSCLC cells, because able to regulate the *Bcl-x* cytoplasmic expression level and thus apoptotic and autophagic processes [162].

## 13. Selenium

Selenium (Se) is an essential element for humans, plants and microorganisms, naturally present and mainly diffused in two inorganic forms, selenite (Se4+) and selenate (Se6+), as well as in their organic derivatives. In spite of the other selenium compounds, usually associated with an antioxidant activity, sodium selenite is an oxidizing agent that makes cancer cells more prone to oxidative stress. Several studies showed, indeed, its cytotoxic properties, through direct or indirect activation of natural killer (NK) cells and by inhibiting the disulfide exchange on the surface of cancer cell membranes (phenomenon usually related to the uncontrolled cell division) and inducing changes in the structure of proteins required for cell survival, it makes cancer cells more susceptible to the activity of phagocytic cells and to the apoptotic mechanism [163,164].

The use of selenite, therefore, seems to exhibit promising anticancer effects, as described in numerous studies, also in association with RT.

Schueller et al. showed as 14 days pre-treatment of C6 rat glioma cell line with different selenite concentrations (2–3.6 µM), before γ-rays (0–20 Gy), led to a lower plating efficiency, especially for radiation doses >2 Gy, and to an overall lower survival than the untreated control. In particular, for Se non-toxic concentrations of 0, 2 and 3 µM, respectively, SF2 amounted to 0.72, 0.48 and 0.46 and SF5 to 0.37, 0.25 and 0.12, with an associated D0 value of 6.1, 4.7 and 3.8 Gy) [165].

Other researchers evaluated the effects of selenium, in the vehiculated form of selenium nanoparticles (Nano-Se), as radiosensitizer. Various concentrations of Nano-Se (0–3 µg/mL), for 24 h of treatment, were used before X-rays (0–8 Gy) on MCF-7 breast cancer cells, and radiosensitivity was evaluated with different essays. Combined treatment lead to a higher mortality rate than both treatment used alone (IR or Nano-Se), with a reduction in the colony formation rate to 25.27 and 15.97 under 4 Gy IR associated to 0.15 or 0.3 µg/mL Nano-Se, and to 6.81 and 4.06 under 6 Gy IR associated to 0.15 or 0.3 µg/mL Nano-Se, respectively. Combined treatment, furthermore, lead to an acceleration through G1/S phase inducing cell cycle arrest at the G2/M phase, to the activation of autophagy by the increase of *LC3* positive structures and to an increase in both endogenous and irradiation-induced ROS formation [166].

The radiosensitizing effect of selenium was also observed in A375 human melanoma cells. In particular, Liua et al. studied the effects of a highly hemocompatible erythrocyte membrane-coated ultrasmall selenium nanosystem combined with bevacizumab (RBCs@Se/Av) (0–15 µM; 4 h) combined to X-rays (2–8 Gy). Experimental data showed a strong reduction in the survival fraction of A375 cells after combined treatment (17.5%) compared to X-rays or RBCs@Se/Av alone (56.2% and 96%, respectively), and an increase in the Sub-G1 cell proportion, in the levels of activated *caspases-3/-8/-9* and *PARP* cleavage, demonstrating an increase in the caspase-mediated apoptotic pathway. In addition, this treatment leads to an increase in ROS generation, ROS-mediated mitochondrial fragmentation, in the Ser15 phosphorylated *p53* form and in the levels of many DNA damage markers, but a reduction in the expression level of *VEGF* and *VEGF2*, index of a decrease of tumor angiogenesis [167].

## 14. Discussion

Today is known that the concept that “one size fits for all”, is not applicable in cancer care because patients could be erroneously treated using general therapeutic criteria that did not take into account the complex heterogeneity of cancer. Thus, the finding of the right cure for specific neoplasm represents one of the trickiest challenges of science.

Nowadays, thanks to the introduction of sequencing and gene expression profile techniques, we are conscious about the importance of more and more customized therapies, in fact, tumors affecting the same organ can be further classified into subgroups with a specific biological profile that gives them the ability to acquire resistance to those treatments that are instead effective for tumors with similar histological features.

Fortunately, decades of never-ending research in the field of cancer have led to the establishment of more and more effective therapies aimed to personalize medicine in light of the features both of the cancer and the patient to be treated. Clinical approaches such as chemotherapy and RT have been implemented, while the first has been characterized by the discovery of new and more effective drugs, the latter is based on the same basics since the discovery of radioactivity and its first application in cancer treatment which occurred in 1896 [168].

In respect to chemotherapy, RT is able to deliver a certain dose directly to the tumor, limiting damages to the normal tissue. This ability to spare healthy areas surrounding the tumor is more precise when hadron therapy is used. Due to their nature, charged particles such as protons and carbon ions can deposit most of their energy within the target with little diffusion [169,170].

Both conventional RT, which uses photons (gamma or X-rays), and hadron therapy, are capable to induce cell death because they alter the DNA structure of their targets. DNA breaks can be induced in a direct way when the IR impacts with the double-strand helix or can be defined as indirect when caused by the effects of ROS production triggered by RT. ROS are not only responsible for DNA damage, but they can also oxidize almost every molecular structure within the cells, causing their death [171].

However, as it often occurs with chemotherapy, cancer can also acquire resistance to RT. Therefore, the administration of radiosensitizing compounds could coadjuvate RT itself. Nowadays, several drugs are used with this purpose and they are commonly referred to as hypoxic and non-hypoxic radiosensitizer (e.g., nitroimidazoles and halogenated pyrimidines) but they yet exhibit collateral systemic effects. The use of nutraceuticals, which may mimic the effect of chemically synthesized radiosensitizer, could help to overcome this issue since they are characterized by low toxicity. Recently, the interest in natural compounds for the treatment of several pathologies has risen, this is not only due to their less detrimental effects but also because of their low economical costs.

The health-promoting effects of compounds coming from nature have been known for thousands of years and their use in medical care still plays a pivotal role in traditional medicines, such as Chinese and Ayurvedic traditional medicine. The beneficial effects of nutraceuticals are exploited in hypertension, diabetes, osteoporosis and lipid control and they have been introduced in clinical practice as neo-adjuvant of chemotherapy [172,173,174].

**Figure 1:**
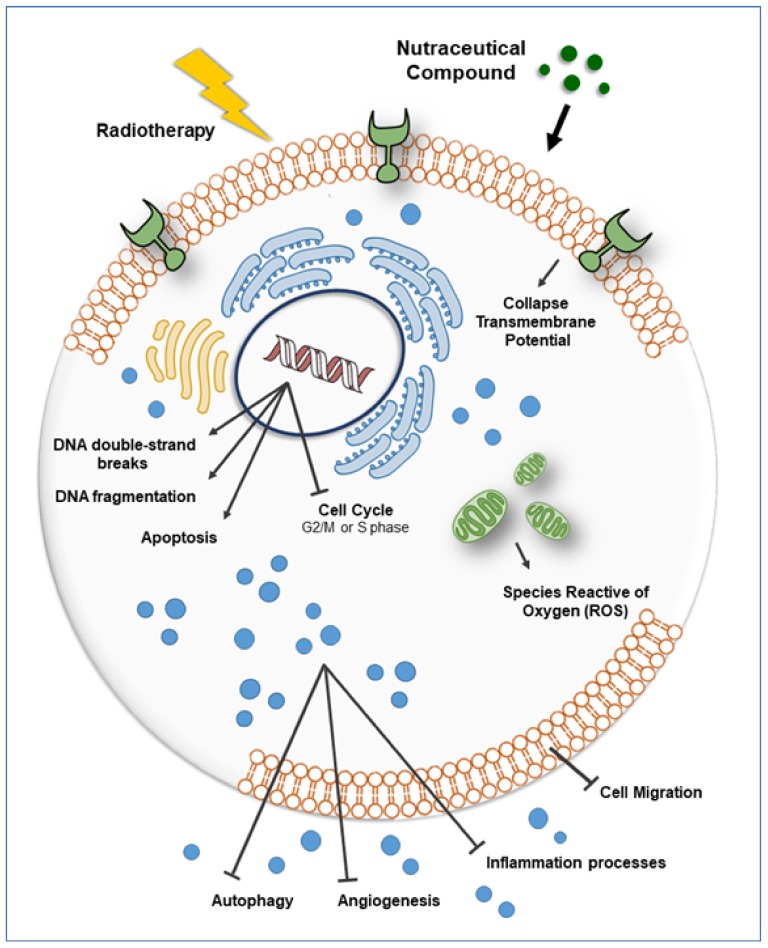
The figure displays how nutraceuticals compounds and radiation treatment could affect cellular pathways involved in migration, inflammation, autophagy and reactive oxygen species (ROS) production.

On these bases, it is not hard to expect that nutraceuticals play a role also as mediators of radiosensitization. According to the numerous scientific papers describing their anticancer activity, we tried to collect data regarding the most known natural compounds: Curcumin, Resveratrol, Withaferin, Celastrol, Ursolic Acid, Zerumbone, CAPE, Emodin, Flavopiridol, Berberine, Genistein and Selenium, which demonstrate their beneficial effects even as radiosensitizers (Table 1). All the results we analyzed share a unique leitmotif, and apart for few nutraceuticals taken into exams, the best characterized molecular effects involved in radiosensitization of tumors are: The activation of pro-apoptotic signals as demonstrated by their overall ability to induce downregulation of *BCL-2*, increase of *PARP* and *Caspase-3* cleavage; a wide-ranging increase of cells blocked in the G2/M cell cycle phase which is, in fact, the most responsive stage of mitosis to IR; a frequent inhibition of *HIF-1α* and *VEGF* and thus of tumor angiogenesis. Other and less studied pathways induced by nutraceuticals such as cell migration, inflammation, autophagy and ROS production, have been summarized in Figure 1.

Moreover, many of the mechanisms underlying the protective effects of nutraceuticals has not been clarified and are yet to be described. A further and deeper understanding of the key mechanisms involved in radiosensitization driven by nutraceuticals could give a clearer picture of the pathways affected by their activity and would help to identify new targets to increase cell radiosensitization.

## 15. Conclusions

Unlike chemotherapy, recently based on the development of new drugs able to interfere with specific tumor targets, RT is less reliant on the biological features of cancer to be treated. As a matter of fact, each cancer (even the ones affecting the same organ) exhibits distinctive characteristics that are tied with a different RT response and degree of relapses after RT. An increase in terms of response to treatment can be reached thanks to the synergistic effects given by the administration of radiosensitizing compounds that have been lately introduced in clinical practice as neoadjuvant for RT. However, synthetic radiosensitizers show collateral effects that exacerbate the ones already caused by RT. For this reason, the use of nutraceuticals which can counteract the mechanisms of tumor resistance to RT but still with less collateral effects are the topic of several scientific projects aimed to test their efficacy.

**Figure 2:**
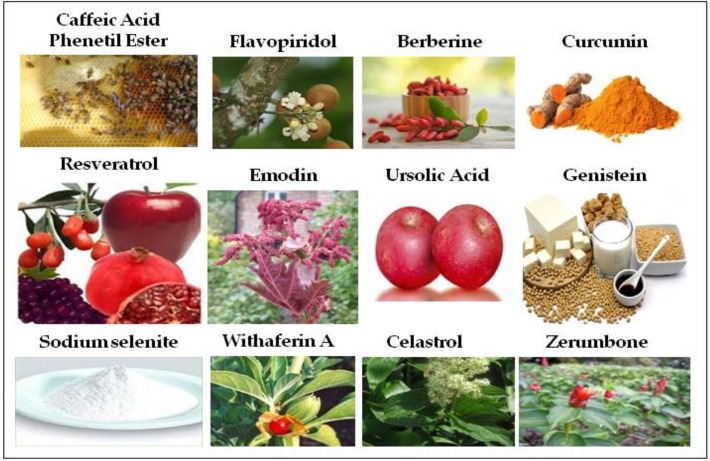
The figure displays the main sources of natural compounds cited in this review.

The focus of our review is to propose an overview of the state of the art of the adoption of nutraceuticals as adjuvant in RT (Figure 2) and to give some hints about the potential pathways involved in their activity.

## Figures and Tables

**Table 1:** The table shows the most relevant and updated works regarding the radiosensitizer effect of the most known natural compounds, cited in this review.

Nutraceuticals	Structure and Molecular Formula	Tumor Targets	Type of Treatment	Bibliography
Curcumin	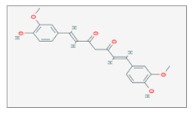 C_21_H_20_O_6_	Breast cancer, Colonrectal Cancer, Glioblastoma Multiforme, Head and Neck squamous Cancer, Prostate Cancer.	X-rays	[14,15,16,17,18]
Resveratrol	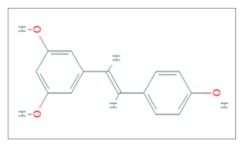 C_14_H_12_O_3_	Breast Cancer, Glioblastoma, Head and Neck squamous Cancer, Melanoma, Nasopharyngeal Carcinoma, Non-Small Cell Lung Cancer, Prostate Cancer.	γ-rays X-rays	[25,26,27,28,29,30,31,32]
Withaferin A	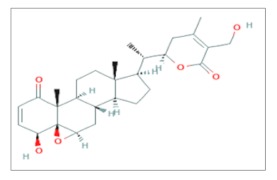 C_28_H_38_O_6_	Breast Cancer, Cervical Cancer, Ehrlich Ascites Carcinoma, Fibrosarcoma, Histiocytic Human Lymphoma, Liver Cancer, Melanoma, Renal Carcinoma.	γ-rays X-rays	[37,38,39,40,41,42,43]
Celastrol	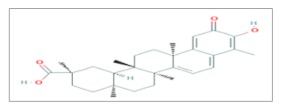 C_29_H_38_O_4_	Lung Cancer, Prostate Cancer.	γ-rays X-rays	[45,47,48]
Ursolic Acid	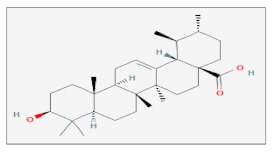 C_30_H_48_O_3_	Colon Carcinoma, Gastric Adenocarcinoma, Melanoma, Non-Small Cell Lung Cancer, Prostate Cancer.	γ-rays X-rays	[50,51,52,53]
Zerumbone	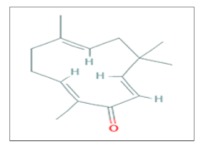 C_15_H_22_O	Colonrectal Cancer, Glioblastoma, Lung adenocarcinoma, Non-Small Cell Lung Cancer, Prostate Cancer.	γ-rays X-rays	[60,62,63,64]
Caffeic Acid Phenetyl Ester	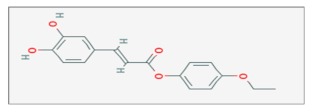 C_17_H_16_O_5_	Adenocarcinoma, Breast Cancer, Lung Cancer, Medulloblastoma.	γ-rays X-rays	[68,69,70,71,72]
Emodin	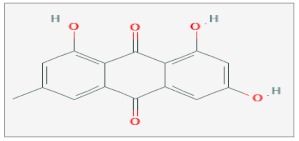 C_15_H_10_O_5_	Cervical Cancer, Hepatocellular Carcinoma, Nasopharyngeal Carcinoma, Sarcoma.	γ-rays X-rays	[79,80,81,82,83]
Flavopiridol	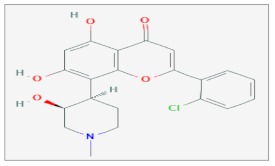 C_21_H_20_ClNO_5_	Cervix Cancer, Esophageal adenocarcinoma, Esophageal squamous Carcinoma, Glioma, Lung Carcinoma, Ovarian Carcinoma, Prostate Cancer, Zebrafish Model.	γ-rays X-rays	[91,92,93,94,95,101,109,110,111,112].
Berberin	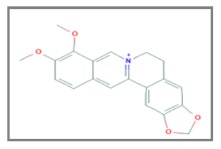 C_20_H_18_NO_4_	Breast Cancer, Esophageal Carcinoma, Lung Carcinoma, Nasopharyngeal Carcinoma, Osteosarcoma, Prostate Cancer.	γ-rays X-rays	[122,123,124,127,128,134,140]
Genistein	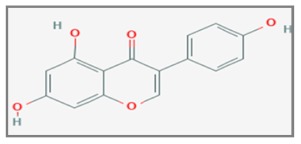 C_15_H_10_O_5_	Breast Cancer, Cervical Cancer, Non-Small Cell Lung Cancer.	γ-rays X-rays	[148,150,151,152,158,162]
Sodium Selenite	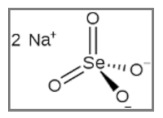 NaSeO_3_	Breast Cancer; Glioma; Melanoma.	γ-rays X-rays	[165,166,167]
